# Optimizing Cardiovascular Health: A Comprehensive Review of Risk Assessment Strategies for Primary Prevention

**DOI:** 10.7759/cureus.66341

**Published:** 2024-08-06

**Authors:** Vineet Karwa, Anil Wanjari, Sunil Kumar, Rushikesh H Dhondge, Rajvardhan Patil, Manjeet Kothari

**Affiliations:** 1 Department of Internal Medicine, Jawaharlal Nehru Medical College, Datta Meghe Institute of Higher Education and Research, Wardha, IND

**Keywords:** pharmacological interventions, lifestyle modifications, risk factors, primary prevention, risk assessment, cardiovascular disease

## Abstract

Cardiovascular disease (CVD) is a leading global health concern, and effective primary prevention strategies are essential to mitigate its impact. This comprehensive review examines current risk assessment strategies for primary prevention of CVD, emphasizing the importance of early identification and intervention to reduce disease incidence. Traditional risk factors such as hypertension, hyperlipidemia, smoking, and lifestyle choices are discussed alongside emerging factors, including genetic predispositions and biomarkers. The review evaluates various risk assessment tools and models, such as the Framingham risk score, atherosclerotic CVD risk calculator, QRISK, and Reynolds risk score, highlighting their methodologies, strengths, and limitations. Additionally, the review explores lifestyle modifications, including dietary changes, physical activity, weight management, smoking cessation, and pharmacological interventions like statins and antihypertensives. Special considerations for different populations, including the elderly, women, and those with a family history of CVD, are addressed. Future directions in cardiovascular risk assessment are also discussed, focusing on technological advancements and personalized medicine. This review aims to enhance the implementation of effective primary prevention measures and improve cardiovascular health outcomes by providing a thorough analysis of risk assessment strategies.

## Introduction and background

Cardiovascular disease (CVD) remains one of the leading causes of morbidity and mortality worldwide [1]. Conditions such as coronary artery disease (CAD), stroke, and hypertension significantly contribute to this burden, underscoring the importance of effective strategies to prevent their onset. Primary prevention is crucial in this context, focusing on reducing the risk of CVD before symptoms appear [2]. This approach involves targeting modifiable risk factors, including hypertension, hyperlipidemia, smoking, and poor lifestyle choices, to prevent the progression to symptomatic disease. By addressing these factors proactively, primary prevention aims to reduce the overall incidence of cardiovascular events and improve public health outcomes [3].

Risk assessment is a pivotal element in the primary prevention of CVD, as it enables healthcare providers to identify individuals at an increased risk of developing these conditions [4]. Accurate risk assessment allows for the early implementation of preventive measures and interventions tailored to an individual's risk profile. Various risk assessment tools and models have been developed to estimate cardiovascular risk, incorporating age, gender, blood pressure, cholesterol levels, and lifestyle habits [5]. These tools help in stratifying risk, facilitating personalized prevention strategies that enhance the effectiveness of interventions, and optimizing resource allocation [6].

The primary objective of this comprehensive review is to provide an in-depth evaluation of current risk assessment strategies for the primary prevention of CVD. This review will explore the traditional and emerging risk factors associated with CVD and their implications for risk assessment. It will also assess existing risk assessment tools and models' methodologies, strengths, and limitations. Furthermore, the review will discuss lifestyle modifications and pharmacological interventions that can be employed to manage cardiovascular risk effectively. Special populations, which may require tailored risk assessment and prevention approaches, will be highlighted. Finally, the review will explore future directions in cardiovascular risk assessment, considering technological advancements and personalized medicine. This review aims to enhance the understanding and application of risk assessment strategies by addressing these objectives, ultimately contributing to improved primary prevention efforts and cardiovascular health outcomes.

## Review

Overview of cardiovascular risk factors

Traditional Risk Factors

Traditional cardiovascular risk factors include modifiable and nonmodifiable factors that significantly contribute to CVD development. Modifiable factors, such as hypertension, dyslipidemia, diabetes mellitus, tobacco use, obesity, physical inactivity, and an unhealthy diet, can be altered or managed through lifestyle changes and medical interventions. Nonmodifiable factors include age, sex, family history, and race/ethnicity, which are inherent and cannot be changed. Addressing these traditional risk factors is crucial for effective screening and prevention strategies [7]. Modifiable risk factors are those that individuals can change or manage to reduce their risk of CVD. Hypertension, or high blood pressure, is one of the most significant modifiable factors, as it can damage blood vessels and the heart, increasing the risk of heart attacks and strokes [8]. Dyslipidemia is another critical factor contributing to atherosclerosis development, characterized by elevated low-density lipoprotein (LDL) cholesterol and low levels of high-density lipoprotein (HDL) cholesterol. Diabetes mellitus also significantly raises the risk of CVD, as it is often associated with hypertension and dyslipidemia and causes direct damage to blood vessels [9]. Tobacco use is a major preventable cause of CVD, contributing to atherosclerosis and increasing the risk of heart attacks and strokes. Obesity, particularly abdominal obesity, is linked to higher risks of hypertension, diabetes, and dyslipidemia, all of which contribute to CVD. Physical inactivity is associated with an increased risk, as a sedentary lifestyle can lead to weight gain and exacerbate other risk factors. An unhealthy diet, high in saturated fats, trans fats, and sugars, can lead to obesity and dyslipidemia, further increasing the risk of cardiovascular issues [10].

Nonmodifiable risk factors, although unchangeable, are important to consider in risk assessment. Age is a significant factor, with the risk of CVD increasing with advancing age, particularly in men over 45 and women over 55 [11]. Family history also plays a role; individuals with a family history of premature heart disease may have a higher risk, especially if relatives develop heart conditions at a young age. Sex is another factor, with men generally having a higher risk of CVD at a younger age compared to women, although this risk for women increases to the risk of men after menopause. Race and ethnicity can influence risk, as certain groups, such as African Americans and South Asians, have a higher prevalence of CVD and related risk factors [7]. Understanding these traditional risk factors is crucial for effective screening and prevention strategies in clinical practice. Tools like the Framingham risk score (FRS) and pooled cohort equations utilize these factors to estimate an individual's risk for developing CVD, guiding management strategies and interventions to improve cardiovascular health [12].

Emerging Risk Factors

Emerging risk factors for CVD are gaining attention due to their potential to provide additional insights into cardiovascular health beyond traditional risk factors. Understanding these factors is crucial for enhancing risk assessment and prevention strategies. While conventional risk factors such as hypertension, smoking, and diabetes remain important, emerging factors can help refine our understanding of individual risk profiles [13]. One significant emerging risk factor is the coronary artery calcium (CAC) score. This imaging technique quantifies calcified plaque in the coronary arteries and strongly predicts future cardiovascular events. Elevated CAC scores indicate a higher risk of CAD and can assist in refining risk stratification, particularly in patients with intermediate risk. Alongside CAC, C-reactive protein (CRP) levels are also recognized as important. CRP is a marker of systemic inflammation, and high levels have been associated with increased cardiovascular risk, indicating underlying inflammatory processes that contribute to atherosclerosis [14].

Another emerging factor is apolipoprotein B (ApoB), which reflects the number of atherogenic particles in the bloodstream. Research suggests that ApoB may better predict cardiovascular risk than traditional lipid measures alone. Similarly, lipoprotein(a), a genetically determined lipoprotein variant, has been linked to an increased risk of atherosclerosis and cardiovascular events. Its measurement is becoming more common in risk assessment, particularly for individuals with a family history of premature heart disease [15]. Carotid intima-media thickness (CIMT) is another important emerging risk factor. This ultrasound measure assesses the thickness of the carotid artery walls, with increased CIMT associated with a higher risk of cardiovascular events. It can be used to identify subclinical atherosclerosis, providing valuable information for early intervention. Additionally, elevated homocysteine levels, an amino acid linked to endothelial dysfunction and vascular injury, have been associated with an increased risk of CVD. However, its role as a direct risk factor remains debated [16].

Psychosocial factors are also gaining recognition as significant contributors to cardiovascular risk. Conditions such as depression, anxiety, and chronic stress can lead to unhealthy behaviors and physiological changes that elevate cardiovascular risk. Moreover, pregnancy-related complications such as gestational diabetes and preeclampsia have been associated with a higher risk of developing CVD later in life for women, highlighting the importance of monitoring cardiovascular health in this population [17]. Environmental factors, including low socioeconomic status and exposure to air pollution, have also been identified as risk factors for CVD. These factors can contribute to increased stress and limited access to healthcare, exacerbating cardiovascular risk. Finally, genetic predispositions, including variations in genes related to lipid metabolism and inflammation, can influence individual susceptibility to CVD, underscoring the need for personalized prevention strategies [18].

Risk assessment tools and models

Framingham Risk Score

The FRS is a widely utilized tool designed to estimate an individual's 10-year risk of developing CVD. This scoring system was developed based on data from the Framingham Heart Study, a long-term cohort study that has tracked participants since 1948 [19]. The FRS specifically estimates the risk of "hard" coronary heart disease (CHD) events, such as myocardial infarction and coronary death, in men and women aged 30-74 years who do not have known CVD [20]. The methodology assesses key risk factors, including age, sex, smoking status, blood pressure, cholesterol levels, and diabetes. Each risk factor is assigned a certain number of points based on its contribution to overall cardiovascular risk, and the total points accumulated correspond to a percentage that indicates the 10-year risk [21]. One of the primary strengths of the FRS is its widespread use and validation across various populations. As one of the first tools developed for cardiovascular risk assessment, it provides a quantitative estimate that can significantly aid healthcare providers in guiding prevention strategies and treatment decisions. The FRS has also been adapted for use in different clinical settings, making it a versatile tool for risk assessment. Its straightforward methodology allows for easy implementation in practice, providing clinicians with a clear framework for identifying individuals at a higher risk of CVD [22]. However, the FRS is not without its limitations. One notable concern is that it may overestimate risk in low-risk populations while underestimating risk in high-risk groups, such as those with socioeconomic deprivation or specific health conditions. Additionally, the FRS focuses primarily on "hard" coronary events and does not account for other cardiovascular outcomes, such as stroke or heart failure [23]. Furthermore, the risk factors used in the FRS are typically measured at a single point, which may not accurately reflect changes in an individual's health status over time. These limitations highlight the need for ongoing research and the development of more comprehensive risk assessment tools to better account for cardiovascular health's complexities across diverse populations [24].

ASCVD Risk Calculator

The atherosclerotic cardiovascular disease (ASCVD) risk calculator, developed by the American College of Cardiology (ACC) and the American Heart Association (AHA), is a widely used tool designed to estimate an individual's 10-year risk of developing ASCVD. This category of disease encompasses serious conditions such as coronary death, nonfatal myocardial infarction, and both fatal and nonfatal strokes. The calculator is based on the pooled cohort equations, which draw from data collected across multiple community-based populations, ensuring a broad and representative foundation for its risk estimations [25]. To use the ASCVD risk calculator, healthcare providers input several key variables, including the patient's age, sex, race, total cholesterol levels, HDL cholesterol levels, systolic blood pressure, whether the patient is taking blood pressure-lowering medication, diabetes status, and smoking status. This methodology allows for a comprehensive cardiovascular risk assessment tailored to individual patient profiles. The tool is specifically applicable to African American and non-Hispanic White men and women aged 40-79 years, making it a critical resource in primary care settings for identifying individuals who may benefit from preventive interventions [26]. Despite its strengths, the ASCVD risk calculator has notable limitations. While it provides a quantitative estimation of absolute risk based on group averages, it does not predict individual outcomes with precision. Furthermore, the calculator may underestimate risk for certain ethnic groups, such as American Indians, South Asian Americans, and Puerto Ricans, while potentially overestimating risk for East Asian Americans and Mexican Americans. Additionally, it is important to note that the calculator is intended solely for primary prevention and does not apply to individuals who already have established ASCVD. Therefore, healthcare providers should use the calculator with clinical judgment and consider patient preferences when developing prevention strategies [26].

QRISK

QRISK is a widely used algorithm designed to estimate an individual's 10-year risk of experiencing a heart attack or stroke [27]. Developed by doctors and academics, QRISK utilizes data from thousands of general practices across the United Kingdom, specifically through the QResearch database. The methodology behind QRISK involves sophisticated statistical techniques, including fractional polynomials, which allow for the modeling of nonlinear relationships between risk factors and cardiovascular events [28]. Additionally, multiple imputation methods are employed to handle missing data, thus minimizing potential biases. The algorithm is updated annually by ClinRisk Ltd., London, UK, to ensure that it reflects changes in population characteristics and improvements in data quality, making it a relevant tool for contemporary clinical practice [29]. One of the key strengths of QRISK is its comprehensive approach to assessing cardiovascular risk. The algorithm incorporates many traditional risk factors, such as age, sex, cholesterol levels, blood pressure, and smoking status [28]. Moreover, it includes additional variables like body mass index, family history of CVD, social deprivation, and the use of antihypertensive treatment. This extensive inclusion of risk factors enhances the accuracy of risk predictions. QRISK has also undergone rigorous validation in independent UK primary care databases, such as the Clinical Practice Research Datalink and The Health Improvement Network, as well as in various clinical cohorts. Research indicates that QRISK often outperforms the Framingham risk equation, particularly in identifying high-risk individuals within the UK population [30]. Despite its strengths, QRISK has several limitations that warrant consideration. One notable concern is that the algorithm is not based on a randomly selected cohort, which raises questions about its representativeness of the broader UK population. Additionally, the risk factors used in the model were measured at varying times relative to study entry, which may affect the accuracy of risk assessments [28]. There is also limited information regarding the standardization of risk factor measurements across different practices. A significant challenge is the high rate of missing data for key risk factors, such as cholesterol ratios, which can affect the reliability of the risk estimates. Furthermore, the hazard ratio for cholesterol ratios derived from QRISK is inconsistent with findings from previous studies, potentially due to the methods used to handle missing data. Finally, while QRISK may underestimate 10-year cardiovascular risk, this underprediction is generally smaller than the overprediction observed with the Framingham model [31].

Reynolds Risk Score

The Reynolds risk score (RRS) is a risk assessment tool designed to predict the 10-year risk of cardiovascular events, particularly in women. This score incorporates both traditional and novel risk factors to enhance the accuracy of cardiovascular risk prediction [32]. Key components of the RRS include age, systolic blood pressure, total cholesterol, HDL cholesterol levels, high-sensitivity C-reactive protein (hsCRP), smoking status, and family history of premature myocardial infarction. The RRS was developed using data from a large cohort of initially healthy women, focusing on identifying those at higher risk for cardiovascular events such as myocardial infarction and stroke over 10 years. The model was validated against observed outcomes to ensure its predictive accuracy [33]. One of the primary strengths of the RRS is its inclusion of novel biomarkers, particularly hsCRP, which reflects inflammation and is associated with cardiovascular risk. This addition allows for a more comprehensive assessment than traditional models like the FRS [34]. Moreover, the RRS is specifically tailored for women, addressing a significant gap in cardiovascular risk assessment that often relied on male-centric data. By integrating traditional risk factors and family history, the RRS provides a holistic view of an individual's cardiovascular risk profile, which can be particularly beneficial in clinical decision-making [35]. However, the RRS also has limitations. Notably, it is not applicable for individuals with diabetes, which excludes a significant patient population that is at high risk for cardiovascular events. Additionally, the score's complexity and multiple variables may complicate its application in clinical settings, especially in primary care, where simpler tools might be preferred. Furthermore, while the RRS has been validated in a specific cohort, its applicability across different ethnicities and populations may require further validation to maintain predictive accuracy in diverse groups [36].

Other Emerging Tools

Emerging cardiovascular risk assessment tools enhance the ability to predict and manage CVD more effectively. Two significant development areas include using biomarkers and advanced imaging techniques [37]. Biomarkers are increasingly recognized for their potential to improve cardiovascular risk assessment. Among these, polygenic risk scores have gained attention for their ability to aggregate the effects of multiple genetic variants, providing an estimate of an individual's risk for developing CVD. This approach is particularly valuable in younger patients or those with a family history of premature cardiovascular events, as it can identify individuals who may benefit from early intervention. Additionally, metabolic risk scores evaluate metabolic factors such as insulin resistance and lipid profiles, offering a more nuanced understanding of an individual's risk beyond traditional cardiovascular risk factors. These biomarkers can help clinicians tailor prevention strategies more effectively [38]. Advanced imaging techniques are also playing a crucial role in cardiovascular risk assessment. One notable method is CAC scoring, a noninvasive imaging technique that quantifies the amount of calcium in the coronary arteries [39]. This measurement serves as a marker for subclinical atherosclerosis and is particularly useful for risk stratification in patients with borderline to intermediate 10-year ASCVD risk. Research has shown that CAC scoring can improve risk prediction and better guide preventive strategies than traditional risk factors alone. Other advanced imaging modalities, such as cardiac MRI and computed tomography (CT) angiography, are being explored for their potential to visualize and quantify cardiovascular risk factors, providing additional insights into vascular health and plaque characteristics [40].

Lifestyle modifications for cardiovascular health

Dietary Changes

Diet plays a crucial role in influencing cardiovascular health. A healthy diet can significantly reduce the risk of CVD by affecting various risk factors, including blood pressure, blood lipids, obesity, inflammation, and endothelial function. Evidence from prospective cohort studies indicates that dietary patterns rich in fruits, vegetables, whole grains, and healthy fats are associated with lower CVD risk. Conversely, diets high in saturated fats, refined carbohydrates, and processed meats are linked to increased CVD risk [41]. Research has shown that specific dietary changes can lead to substantial health benefits. For instance, increasing fruit and vegetable intake has been consistently associated with a reduced risk of CHD and stroke. A meta-analysis indicated that higher consumption of fruits and vegetables correlates with a significant decrease in stroke risk. At the same time, other studies suggest that replacing red and processed meats with healthier protein sources like fish, nuts, and legumes can lower overall CVD mortality. Overall, the evidence underscores the importance of dietary choices in managing and preventing CVD [42].

The Mediterranean diet is widely recognized as particularly beneficial for cardiovascular health among the various dietary patterns. This diet emphasizes high consumption of plant-based foods, including fruits, vegetables, whole grains, legumes, and nuts. It encourages olive oil as the primary fat source, rich in monounsaturated fats known for their heart-protective properties. The Mediterranean diet also recommends moderate intake of fish and poultry, highlighting the importance of oily fish due to their omega-3 fatty acids, which are protective against heart disease. In contrast, it advises limiting red and processed meat, promoting low-fat dairy products, and allowing moderate alcohol consumption, primarily red wine [43]. In addition to the Mediterranean diet, other dietary patterns have shown promise in reducing CVD risk. The dietary approaches to stop hypertension diet focuses on reducing sodium intake while increasing the consumption of fruits, vegetables, whole grains, and low-fat dairy products, making it effective for managing blood pressure. Plant-based diets, which emphasize whole plant foods while minimizing animal products, can lower cholesterol levels and improve heart health. The flexitarian diet, a primarily vegetarian approach that allows for occasional meat and fish, also promotes a high intake of plant foods while remaining flexible [44].

Physical Activity

Regular physical activity offers many benefits for cardiovascular health, making it a cornerstone of preventive care. Engaging in exercise helps lower blood pressure and rest the heart rate, reducing the overall workload on the heart. Improved circulation and enhanced oxygen delivery to tissues are additional advantages, leading to better cardiovascular function. Moreover, physical activity plays a crucial role in modifying lipid profiles by increasing HDL cholesterol, often referred to as "good" cholesterol, while simultaneously lowering LDL cholesterol, or "bad" cholesterol [45]. Beyond lipid management, exercise is instrumental in controlling blood sugar levels and reducing the risk of developing type 2 diabetes, a significant risk factor for CVD. It also aids in maintaining a healthy body weight and reduces systemic inflammation, both critical for heart health. Furthermore, regular exercise strengthens the heart muscle, improving cardiac output and efficiency. Research consistently shows that physically active individuals have a lower risk of cardiovascular events, such as heart attacks and strokes, compared to sedentary individuals, even when controlling for other risk factors like smoking and obesity [46].

Health organizations such as the AHA recommend specific exercise guidelines to harness the cardiovascular benefits of physical activity. Adults should aim for at least 150 minutes of moderate-intensity aerobic activity each week, including brisk walking or 75 minutes of vigorous-intensity aerobic exercise, such as jogging or swimming. This translates to approximately 30 minutes of aerobic activity on most days of the week [47]. In addition to aerobic exercise, it is essential to incorporate muscle-strengthening activities that engage all major muscle groups at least two days per week. Combining aerobic exercise with resistance training maximizes cardiovascular benefits and promotes physical fitness. Flexibility and balance exercises should also be included regularly to support musculoskeletal health and facilitate participation in other forms of exercise [48]. Importantly, even modest amounts of physical activity can yield significant cardiovascular benefits, and the health gains increase with higher activity levels. However, it is crucial to note that excessive exercise, such as training for marathons, may have potential risks for some individuals. Therefore, it is advisable to consult with a healthcare provider to establish an appropriate exercise regimen tailored to one's health status and fitness goals. By following these guidelines, individuals can significantly enhance their cardiovascular and overall well-being [49].

Weight Management

Obesity is increasingly recognized as a significant risk factor for CVD, contributing to its development through various pathophysiological mechanisms. The expansion of visceral adipose tissue leads to dysregulation of adipokines, insulin resistance, endothelial dysfunction, and a pro-inflammatory state, all elevating susceptibility to CVD events [50]. A primary mechanism linking obesity to CVD is adipokine dysregulation. In individuals with obesity, the secretion of adipokines, hormones produced by adipose tissue, is altered, promoting inflammation and insulin resistance. This dysregulation triggers a cascade of metabolic disturbances that adversely affect cardiovascular health. Additionally, obesity contributes to endothelial dysfunction, impairing blood vessels' ability to dilate and increasing the risk of hypertension and atherosclerosis [51]. Chronic inflammation is another critical factor in the relationship between obesity and CVD. Excess adipose tissue produces inflammatory cytokines, creating a state of chronic low-grade inflammation that contributes to atherosclerosis and other cardiovascular conditions. Moreover, obesity is a core component of metabolic syndrome, which includes hypertension, dyslipidemia, and type 2 diabetes, further increasing the risk of cardiovascular events. Interestingly, some studies have observed an "obesity paradox," where individuals with obesity may experience better short-term outcomes after cardiovascular events. This phenomenon may be due to earlier diagnosis and treatment in obese patients and differences in fitness levels [52]. Achieving and maintaining a healthy weight is crucial for reducing CVD risk. Effective weight management strategies include lifestyle modifications, medical interventions, and long-term maintenance [53].

Lifestyle modifications form the foundation of effective weight loss. A balanced diet of fruits, vegetables, whole grains, and lean proteins can facilitate weight loss. Diets such as the Mediterranean and plant-based diets have demonstrated positive effects on weight management and cardiovascular health. Regular physical activity is also essential; the Centers for Disease Control and Prevention recommends at least 150 minutes of moderate-intensity aerobic activity each week, combined with strength training exercises two or more days per week. Incorporating behavioral strategies, such as goal setting, self-monitoring, and seeking social support, can further enhance adherence to weight loss programs [54]. Medical interventions may be appropriate for individuals who struggle to lose weight through lifestyle changes alone. Pharmacotherapy, including appetite suppressants and medications that affect metabolic pathways, can assist in weight management. In more severe cases of obesity, bariatric surgery may be considered. This surgical option can lead to significant weight loss and improvements in cardiovascular risk factors, with studies indicating that surgical patients experience lower rates of cardiovascular events compared to those who only pursue lifestyle changes [55]. Long-term maintenance of weight loss is critical for sustaining health benefits. A weight loss of 5%-10% can lead to significant improvements in health. In addition, maintaining a loss of 10%-15% or more can further enhance cardiovascular health. Continuous monitoring and support from healthcare providers can help individuals maintain their weight loss and effectively manage CVD risk factors. In summary, addressing obesity through a comprehensive approach that includes lifestyle changes, medical interventions, and ongoing support is vital for reducing the risk of CVD and improving overall health outcomes [56].

Smoking Cessation

Smoking has a profoundly detrimental impact on cardiovascular health, significantly increasing the risk of morbidity and mortality. As a major cause of CVD, smoking is responsible for one in every four deaths related to heart disease. The harmful substances in cigarette smoke elevate triglyceride levels and reduce "good" cholesterol (HDL), making the blood more prone to clotting. This can lead to blockages in blood flow to the heart and brain, resulting in heart attacks and strokes [57]. Furthermore, smoking accelerates atherosclerosis, where toxic chemicals cause inflammation and plaque buildup in the arteries. This condition narrows and hardens the arteries, restricting blood flow and increasing the likelihood of cardiovascular events. Smoking also contributes to hypertension, as nicotine constricts blood vessels, further elevating blood pressure and the risk of heart disease. Additionally, smoking can trigger arrhythmias, leading to irregular heartbeats and other rhythm disorders that can be life-threatening [58]. The impact of smoking extends beyond the heart, leading to peripheral artery disease, which reduces blood flow to the limbs and can result in severe complications, including amputation. Smoking also weakens the abdominal aorta, increasing the risk of abdominal aortic aneurysms, which can be life-threatening if they rupture. Importantly, quitting smoking can significantly reduce these risks, with health benefits beginning shortly after cessation and continuing to improve over time [59]. Effective smoking cessation strategies are crucial for reducing cardiovascular risks and improving overall health. One of the most impactful approaches is counseling and behavioral support. Engaging with a counselor who specializes in smoking cessation can help individuals develop a personalized quit plan, manage cravings, and cope with stress related to withdrawal. Research indicates that combining counseling with medication often yields the best results, making it a valuable component of any cessation strategy [60].

**Figure 1:**
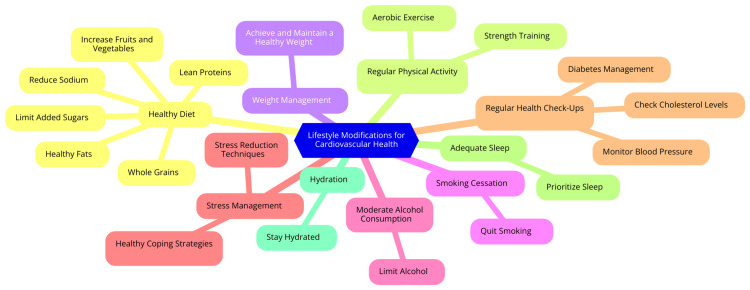
Lifestyle modifications for cardiovascular health Image credit: Vineet Karwa

Pharmacotherapy also plays a significant role in aiding individuals to quit smoking. Various medications, including nicotine replacement therapies (such as patches and gums) and prescription medications like varenicline and bupropion, can assist in reducing withdrawal symptoms and cravings. These medications help individuals manage the physical aspects of quitting, making the process more manageable [61]. Support groups and programs are another effective resource for those looking to quit smoking. Participation in these groups can provide encouragement, accountability, and shared experiences, which can be invaluable during quitting. Many health organizations offer structured programs tailored to assist individuals in quitting smoking, providing a supportive community [62]. In addition to these methods, setting a specific quit date can help individuals prepare mentally and physically for the change. This date is a milestone, allowing individuals to plan for potential challenges. Identifying triggers, situations or emotions that prompt the urge to smoke, is also crucial. By understanding and avoiding these triggers, individuals can maintain their commitment to quitting [63]. Finally, digital and mobile health tools, such as apps and online resources, can provide ongoing support and track progress, making quitting more engaging and accessible. By combining these strategies, individuals can significantly enhance their chances of successfully quitting smoking and, in turn, improve their cardiovascular health [63]. Lifestyle modifications for cardiovascular health are shown in Figure 1.

Pharmacological interventions

Statins

Statins are a class of medications primarily used to lower cholesterol levels in the blood and reduce the risk of CVD. Their effectiveness and safety profile make them a cornerstone in managing dyslipidemia and preventing heart-related events. Statins function by competitively inhibiting the enzyme 3-hydroxy-3-methylglutaryl-coenzyme A reductase, which is essential in the mevalonate pathway for cholesterol synthesis in the liver. By blocking this enzyme, statins reduce the production of mevalonate, leading to decreased cholesterol synthesis. This reduction in hepatic cholesterol levels stimulates the upregulation of LDL receptors on liver cells, enhancing the clearance of LDL from the bloodstream. Consequently, this lowers circulating LDL cholesterol levels, significantly reducing the risk of atherosclerosis and CHD [64]. Beyond their lipid-lowering effects, statins also exhibit pleiotropic effects, which include improving endothelial function, modulating inflammatory responses, maintaining plaque stability, and preventing blood clot formation. These additional benefits enhance their effectiveness in preventing cardiovascular events beyond mere cholesterol reduction [65].

Statins have proven effective in primary prevention, especially in individuals at high risk for cardiovascular events. The Justification for the Use of Statins in Prevention: An Intervention Trial Evaluating Rosuvastatin trial demonstrated that statins could provide substantial benefits even in patients without a history of high cholesterol or heart disease, particularly those with elevated hsCRP levels, indicating inflammation. Clinical guidelines recommend statin therapy for individuals with a history of CVD, high LDL cholesterol levels, diabetes (particularly those aged 40-75), or a calculated 10-year cardiovascular risk of 10% or greater. Overall, statins can reduce the incidence of major cardiovascular events, including myocardial infarction and stroke, making them a critical component of primary prevention strategies [66]. While statins are generally well-tolerated, they can have side effects, including muscle-related symptoms (myopathy and rhabdomyolysis), liver enzyme elevation, gastrointestinal issues, and a slight increase in the risk of developing type 2 diabetes. When prescribing statins, assessing the patient's overall risk profile, potential drug interactions, and individual tolerance to the medication is important. Regular monitoring and patient education regarding side effects are essential to ensure adherence and optimize outcomes [67].

Antihypertensives

Antihypertensive medications are crucial for managing high blood pressure (hypertension), a major risk factor for CVD. These medications are divided into several classes, each with unique mechanisms of action. Their role in primary prevention is vital, as effective blood pressure control can significantly lower the risk of cardiovascular events [68]. Antihypertensive drugs are classified based on their mechanisms of action. Renin-angiotensin system inhibitors, such as angiotensin-converting enzyme inhibitors and angiotensin II receptor blockers, work by either inhibiting the conversion of angiotensin I to angiotensin II or blocking its action at receptor sites, resulting in vasodilation and reduced blood volume. Diuretics, including thiazide diuretics and loop diuretics, enhance diuresis by preventing sodium and chloride reabsorption in the kidneys. Calcium channel blockers, dihydropyridines and nondihydropyridines, inhibit calcium entry into vascular smooth muscle and cardiac tissues, leading to vasodilation and decreased heart rate and contractility. Beta-blockers primarily reduce heart rate and myocardial contractility, while alpha-blockers and vasodilators directly relax vascular smooth muscle, causing vasodilation and decreased blood pressure [69]. Antihypertensives play a key role in primary prevention strategies for CVDs. Effective hypertension management can significantly reduce the incidence of severe cardiovascular events, such as stroke, myocardial infarction, and heart failure. For example, a reduction in blood pressure by just 5 mmHg can decrease stroke risk by approximately 34%, and lowering blood pressure is associated with a 21% reduction in the risk of ischemic heart disease. Proper blood pressure control also helps prevent the onset of heart failure, especially in high-risk populations [70].

Antidiabetic Agents

Antidiabetic agents are crucial in managing cardiovascular risk for individuals with diabetes, particularly type 2 diabetes (T2D). Beyond controlling blood glucose levels, certain classes of these medications offer cardiovascular benefits, making them integral to comprehensive care for diabetic patients [71]. Sodium-glucose cotransporter-2 (SGLT2) inhibitors, such as empagliflozin and canagliflozin, have been demonstrated to reduce the risk of CVD in T2D patients, especially those with existing cardiovascular risk factors. These medications promote glucose excretion through the urine, lowering blood glucose levels and positively affecting weight and blood pressure. Clinical trials have shown that SGLT2 inhibitors significantly decrease the incidence of major adverse cardiovascular events, including heart failure, hospitalization, and cardiovascular mortality [72].

Another significant class of antidiabetic agents is the glucagon-like peptide-1 receptor agonists (GLP-1a), such as liraglutide and semaglutide. These medications enhance insulin secretion in response to meals and help reduce appetite, contributing to weight loss. GLP-1 receptor agonists have also been associated with improved cardiovascular outcomes, including reductions in heart attack and stroke rates. Like SGLT2 inhibitors, GLP-1 receptor agonists have shown consistent cardiovascular benefits in clinical trials, making them a recommended option for patients with T2D at high cardiovascular risk [73]. While metformin is primarily used for glycemic control, it also offers cardiovascular benefits, especially in overweight patients. As the first-line treatment for T2D, metformin is considered safe even in patients with stable heart failure. In contrast, dipeptidyl peptidase-4 inhibitors generally exhibit neutral cardiovascular effects, meaning they do not significantly alter cardiovascular risk. Consequently, they are less preferred than SGLT2 inhibitors and GLP-1 receptor agonists in high-risk patients. Older classes of medications, such as sulfonylureas and thiazolidinediones, have been linked to increased cardiovascular risks and are typically avoided in patients with established CVD [74].

Aspirin

The use of aspirin for CVD prevention, especially in primary prevention, remains a topic of significant debate. This debate largely stems from differing interpretations of clinical trial data and varying guidelines from health organizations. Current recommendations advocate for an individualized approach to aspirin therapy. For adults aged 40-59 years with a 10% or greater 10-year CVD risk, aspirin may be considered. However, for individuals aged 60 and older, the United States Preventive Services Task Force advises against initiating aspirin therapy due to insufficient evidence supporting its benefits relative to the risks of bleeding [75]. In certain populations, such as individuals over 50 with diabetes and additional risk factors, aspirin may still be considered for primary prevention. Conversely, for patients with established heart disease or a history of cardiovascular events, aspirin remains a crucial component of secondary prevention, where its benefits generally outweigh the bleeding risks. Guidelines recommend that healthcare providers conduct a thorough risk-benefit analysis when considering aspirin therapy, as it can reduce the risk of nonfatal myocardial infarction and ischemic stroke but is also associated with an increased risk of major bleeding events [76].

Controversy over aspirin use largely arises from conflicting evidence in recent studies. Meta-analyses have shown that while aspirin reduces the risk of myocardial infarction, it does not significantly lower cardiovascular mortality and may even increase the risk of hemorrhagic stroke. This has sparked debates about the overall efficacy of aspirin in low-risk populations. Additionally, some studies have criticized a uniform approach, suggesting that a tailored strategy based on individual risk factors may be more effective. For example, patients who discontinue aspirin therapy might experience a higher risk of cardiovascular events compared to those who continue its use [77]. Emerging research continues to inform the discussion on aspirin's role in primary prevention. Ongoing studies, such as the Acute Coronary Syndrome Treatment with Angiotensin-Converting Enzyme Inhibitor trial, aim to provide further insights, particularly for specific populations with chronic kidney disease. The findings from these studies are expected to influence future guidelines and recommendations. While aspirin has a well-established role in the secondary prevention of cardiovascular events, its use in primary prevention remains controversial. Healthcare providers must evaluate aspirin therapy case by case, carefully considering individual risk factors, patient preferences, and the evolving evidence base [78].

Integration of risk assessment into clinical practice

Current Guidelines and Recommendations

Current guidelines and recommendations for CVD risk assessment and management emphasize a comprehensive approach to both prevention and treatment. The 2021 European Society of Cardiology (ESC) Guidelines focus on preventing ASCVD and advocate for shared decision-making between patients and healthcare providers. These guidelines are widely endorsed by cardiac societies across Europe, ensuring their broad acceptance and integration into clinical practice. The 2023 ESC Guidelines further refine the management of CVD in patients with diabetes, offering detailed recommendations on risk stratification, screening, diagnosis, and treatment [79]. The 2023 Australian Guideline introduces a new CVD risk calculator tailored to the Australian population, emphasizing early risk factor modification. It provides practical tools for healthcare professionals to assess and manage CVD risk, particularly for First Nations populations. A five-year risk assessment categorizes risk levels into high, intermediate, and low. The December 2023 National Institute for Health and Care Excellence guideline also identifies and assesses CVD risk in adults without established disease. It includes updated recommendations on lipid measurement, statin use for primary and secondary prevention, and lifestyle modifications. This guideline aims to aid healthcare professionals in making informed decisions regarding CVD prevention strategies [80]. The 2019 ACC/AHA Guideline on the primary prevention of CVD underscores the importance of lifestyle factors, such as diet and physical activity, in reducing risk. It incorporates recommendations from previous guidelines on hypertension and cholesterol management, ensuring a holistic approach to primary prevention. In summary, current guidelines emphasize individualized risk assessment, using validated calculators, and integrating lifestyle modifications and pharmacological treatments to effectively manage and prevent CVD. These recommendations are designed to assist healthcare professionals in providing evidence-based care tailored to the needs of diverse patient populations [81].

Implementation Challenges

Integrating cardiovascular risk assessment into clinical practice is crucial for enhancing decision-making and improving patient outcomes. A comprehensive approach involves a structured framework that combines data analysis with clinical judgment, considers the broader organizational context, and incorporates risk management into strategic planning. This approach aims to improve patient safety and healthcare outcomes [82]. However, several challenges can impede the effective integration of cardiovascular risk assessment into clinical practice. Time constraints during patient visits often limit healthcare providers' ability to use cardiovascular risk calculators effectively, as there may not be sufficient time to conduct thorough assessments and communicate risks to patients. Limited access to cardiovascular risk calculators and the necessary information for their effective use, including technological barriers and a lack of guideline-concordant risk communication tools, are also significant obstacles [83]. A lack of enthusiasm or buy-in from healthcare providers and clinic staff can further hinder the adoption of risk assessment tools. When clinicians do not perceive the value or practicality of these tools, they are less likely to incorporate them into their practice. Additionally, patients may fear potential side effects from medications, such as statins, which can discourage them from adhering to recommended preventive measures. This apprehension often arises from a lack of understanding of the benefits versus the risks associated with treatment [84]. The absence of established clinic workflows for using cardiovascular risk calculators can lead to inconsistent application and reliance on traditional assessment methods that do not include risk calculations. Moreover, there is a significant perception gap regarding cardiovascular risk, particularly among women and underserved populations. Many individuals are unaware of their risk factors or the importance of cardiovascular health, leading to lower engagement in preventive care [83]. In older populations, competing mortality risks complicate the interpretation of cardiovascular risk assessments. High-calculated risks may not accurately reflect a patient's overall health status, potentially leading to overmedicalization. Addressing these implementation challenges requires a multifaceted approach, including improving clinician education, enhancing access to tools, fostering patient engagement, and integrating risk assessment into established clinical workflows. Tailoring interventions to overcome these barriers is essential for promoting the effective use of cardiovascular risk calculators in primary care settings [85].

Case Studies and Practical Examples

One notable case study demonstrates the application of a structured cardiovascular risk assessment tool in a primary care setting. A 55-year-old male patient with a family history of heart disease underwent a comprehensive risk evaluation using the FRS. The assessment revealed elevated cholesterol levels and hypertension, placing him at high risk for cardiovascular events. Consequently, the healthcare team implemented a tailored intervention plan, including lifestyle modifications, dietary counseling, and pharmacotherapy. Over the following year, the patient lowered his cholesterol and blood pressure, illustrating how early identification and intervention based on individual risk profiles can improve health outcomes [86]. The British Heart Foundation has compiled a collection of international case studies focusing on innovative strategies for CVD prevention. These studies highlight various interventions across different countries, such as community-based health programs in rural areas and school-based initiatives promoting physical activity among children. For example, a nationwide program in Finland aimed at reducing salt intake and promoting healthy eating has significantly decreased the incidence of heart disease. These case studies showcase the effectiveness of targeted public health initiatives and the importance of adapting strategies to local contexts and populations [87].

A specific case study from the National Institutes of Health explores using the QRISK2 tool, which assesses cardiovascular risk based on age, sex, smoking status, and medical history. In this case, a 60-year-old woman with diabetes was evaluated using QRISK2, which indicated a high risk for cardiovascular events within the next 10 years. This assessment prompted her healthcare provider to initiate a comprehensive management plan, including medication to control her blood sugar and blood pressure, along with lifestyle counseling focused on diet and exercise. The case underscores the utility of risk assessment tools in identifying high-risk individuals and guiding clinical decisions for preventive measures [30]. Another clinical case challenge presented to healthcare professionals involved a 50-year-old man with elevated LDL cholesterol levels and a family history of heart disease. Participants were tasked with assessing their cardiovascular risk and applying evidence-based guidelines for managing hyperlipidemia. Through collaborative discussions, the healthcare team developed a multifaceted approach that included dietary changes, increased physical activity, and statin therapy. This case exemplifies the integration of risk assessment into clinical practice, highlighting the importance of teamwork and adherence to clinical guidelines in optimizing patient care and preventing CVD [88].

Future directions in cardiovascular risk assessment

Advances in Technology and Data Analysis

Technology and data analysis advances significantly transform cardiovascular risk assessment and management, leading to more accurate predictions and personalized care. One of the most notable developments is integrating artificial intelligence (AI) in cardiology. AI algorithms are increasingly employed for predictive analytics, enabling the detection of subtle abnormalities in imaging studies. For example, AI can identify changes in the aortic shape and size during CT scans, potentially flagging life-threatening conditions such as abdominal aortic aneurysms. This capability enhances early diagnosis and intervention, which are critical for improving patient outcomes [89]. Mobile applications are also playing a pivotal role in cardiovascular health management. Innovative tools like the HearO app (Health Innovations Inc., San Francisco, CA) monitor patients' health conditions in real time. By analyzing speech patterns, HearO can detect signs of heart failure, illustrating how technology empowers patients to self-monitor and facilitates early detection of cardiovascular issues. This shift toward patient-centered care is further supported by the development of shared decision-making tools that help patients understand their risks and treatment options [90].

Advances in genetic research are refining cardiovascular risk stratification. Polygenic risk scoring and multiomics approaches are powerful tools that integrate genetic data with traditional risk factors. These methods can identify individuals at higher risk for CVDs earlier in life, allowing for proactive preventive strategies tailored to their unique genetic profiles [91]. Innovations in cardiovascular imaging are also enhancing risk assessment capabilities. Techniques such as coronary computed tomography angiography and CAC scoring provide critical insights into subclinical atherosclerosis, improving risk prediction beyond traditional methods. These advanced imaging modalities enable healthcare providers to visualize the extent of CAD more effectively, facilitating timely and appropriate interventions [92]. The introduction of comprehensive risk calculators, such as the American Heart Association's Predicting Risk of cardiovascular disease EVENT calculator, reflects a growing understanding of the interconnections between various health conditions. This tool combines cardiovascular, kidney, and metabolic health measures to provide a holistic assessment of an individual's risk for cardiovascular events, thereby enhancing clinical decision-making [93]. Finally, wearable technology, including smartwatches and specialized sensors, allows for continuous monitoring of vital signs like heart rate and blood pressure. These devices can transmit real-time data to healthcare providers, enabling timely interventions and personalized care strategies. These technological advancements and data analysis strategies pave the way for more precise, individualized cardiovascular risk assessment and management, ultimately improving patient outcomes [94].

Personalized Medicine and Genomics

Personalized medicine, also known as precision medicine, is an innovative approach that customizes medical treatment based on the individual characteristics of each patient, with a strong emphasis on their genetic profile. This method optimizes therapeutic outcomes by considering genetic, environmental, and lifestyle factors. Personalized medicine uses an individual's genetic information to guide disease prevention, diagnosis, and treatment decisions, shifting away from the "one-size-fits-all" model toward more tailored strategies that enhance treatment efficacy and reduce adverse effects [95]. Genomics is pivotal to personalized medicine. Advanced high-throughput sequencing technologies enable a comprehensive analysis of an individual's genome, allowing healthcare providers to identify genetic predispositions to various diseases, select the most effective medications and dosages based on genetic markers, and develop targeted prevention strategies, especially in oncology and chronic diseases. Pharmacogenomics, a subfield of personalized medicine, focuses on how genetic variations affect a person's response to drugs. By understanding these variations, healthcare providers can tailor drug therapies to improve safety and efficacy, thereby minimizing the risk of adverse drug reactions [96].

The integration of genomic data into routine clinical practice is growing. This includes using molecular profiling tests to guide treatment decisions, incorporating genetic testing into standard care for conditions like cancer and rare genetic disorders, and enhancing screening for hereditary conditions to enable early interventions and preventive measures. Despite its promise, personalized medicine faces several challenges, such as ethical concerns about genetic data privacy and access, comprehensive training for healthcare providers to interpret genomic data effectively, and ensuring equitable access to personalized medicine across diverse populations to prevent worsening health disparities [97]. Future directions in personalized medicine may involve expanding multiomics approaches (integrating genomics, proteomics, and metabolomics) for a more holistic understanding of health and disease, developing AI and machine learning tools to analyze complex genomic data and predict patient outcomes more accurately, and increasing focus on public health initiatives that use genomic data to inform population health strategies and preventive measures. In summary, personalized medicine represents a transformative shift in healthcare, leveraging genomic insights to tailor medical care to individual patients, thereby improving outcomes and enhancing the overall effectiveness of medical interventions [98].

Emerging Research and Innovations

Emerging research and innovations in cardiovascular risk assessment are significantly advancing predictive accuracy, incorporating new technologies, and improving patient outcomes. One of the most promising developments is the identification of novel biomarkers for CVD. Recent studies highlight the critical role of discovering new biomarkers to predict heart disease risk, diagnose conditions, and forecast outcomes. These biomarkers are essential for early detection and management, especially in high-risk populations. Ongoing research is focused on establishing effective single and multimarker strategies to provide comprehensive insights into cardiovascular health [37]. Another exciting advancement is the development of advanced risk assessment techniques, such as polygenic risk scoring and multiomics approaches. These methods refine cardiovascular risk assessment by integrating genetic, metabolic, and clinical data, enhancing risk stratification. This approach is particularly valuable for identifying individuals who might benefit from early interventions, especially when traditional risk factors may not fully capture their risk. The introduction of the quantitative risk assessment 4 (QR4) risk prediction model represents a significant advancement in this field. This tool offers more precise 10-year cardiovascular risk predictions compared to existing models and has been rigorously tested across diverse populations historically underrepresented in risk assessments. The improved predictive capabilities of QR4 could profoundly impact clinical practices and guidelines for cardiovascular health management [99]. Digital health innovations are also revolutionizing cardiovascular risk assessment. The advent of cloud-based e-health systems enables real-time data collection and analysis, providing personalized insights for patients and clinicians. These systems can stratify risks based on various health metrics, supporting a proactive approach to cardiovascular health management. Cardiovascular learning health systems, such as the Utrecht cardiovascular cohort, aim to standardize risk assessments across different clinical specialties. By collecting comprehensive cardiovascular risk profiles, these systems enhance care quality, improve adherence to guidelines, and promote interdisciplinary collaboration among healthcare providers [100].

## Conclusions

In conclusion, optimizing cardiovascular health through effective risk assessment is essential for successfully preventing CVD. The review highlights the critical role of accurate risk assessment in identifying individuals at heightened risk and tailoring preventive strategies to their specific needs. This review provides a comprehensive understanding of mitigating cardiovascular risk by examining traditional and emerging risk factors, evaluating current risk assessment tools, and exploring lifestyle and pharmacological interventions. The discussion extends to special populations, underscoring the necessity for personalized approaches. Looking ahead, advancements in technology and personalized medicine hold promise for further refining risk assessment and prevention strategies. Integrating these insights into clinical practice can enhance the effectiveness of primary prevention efforts, ultimately reducing the incidence of cardiovascular events and improving overall public health. Continued research and innovation are imperative to address existing gaps and develop more precise and individualized cardiovascular risk assessment and management approaches.
